# Management of Bevacizumab-Induced Proteinuria Using an Angiotensin Receptor Blocker (ARB) in a Neurofibromatosis Type 2 (NF-2) Patient With Vestibular Schwannoma

**DOI:** 10.7759/cureus.46202

**Published:** 2023-09-29

**Authors:** Egiroh E Omene, Jay Easaw

**Affiliations:** 1 Medical Oncology, Cross Cancer Institute, Edmonton, CAN

**Keywords:** nf-2, neurofibromatosis type two, arb, unilateral hearing loss, angiotensin receptor blockers, bevacizumab toxicity

## Abstract

Neurofibromatosis type 2 (NF-2) is a genetic condition that by definition includes bilateral vestibular schwannoma, a non-malignant lesion also known as acoustic neuroma. Patients often develop hearing impairment and hearing loss as a result of the involvement of the vestibulocochlear nerve bilaterally as well as attempts at surgical repair. A common treatment for NF-2-mediated schwannoma is the antiangiogenic agent, bevacizumab. In many cases, patients require prolonged and even lifelong treatment with bevacizumab to control schwannoma growth. However, long-term use of bevacizumab can be associated with multiple side effects including hypertension and proteinuria including nephrotic syndrome (>3g of protein excreted in the urine in 24 hours). In these situations, the challenge with discontinuing prolonged bevacizumab can be rapid tumor growth and worsening hearing loss. Pre-clinical data suggests that hearing loss can be prevented following treatment with the angiotensin receptor blocker (ARB), losartan, in an animal model of NF-2. ARBs are already established in nephrology guidelines to treat proteinuria and hypertension. Here, we present a patient with NF-2 who developed nephrotic syndrome while on bevacizumab. Attempts to discontinue bevacizumab resulted in near-immediate hearing loss. Treatment with the ARB telmisartan together with bevacizumab resulted in improved hearing, reduced proteinuria, and controlled hypertension.

## Introduction

Neurofibromatosis type 2 (NF-2) is an autosomal dominant disorder resulting in the eventual development of several types of benign neoplasms within the nervous system. The development of bilateral vestibular schwannoma is so common that it is part of the major diagnostic criteria. While the tumors are benign, their predilection for growing in the vestibulocochlear nerve results in a decreased quality of life for patients because of hearing impairment. Bevacizumab has been used to improve hearing loss and manage tumor growth with clinical trial data demonstrating efficacy in doing so. A consequence of long-term bevacizumab use is nephrotic syndrome with proteinuria. Angiotensin receptor blockade concomitant with bevacizumab may have a synergistic therapeutic effect as seen in metastatic colorectal cancer. This case report illustrates that angiotensin receptor blockade concurrent with bevacizumab can reduce bevacizumab-induced proteinuria and can subjectively improve hearing loss. This hypothesis-generating case report supports the use of angiotensin receptor blockers (ARB) in NF-2 patients treated with long-term bevacizumab. It provides a rationale to investigate the timing of ARB use in NF-2 patients with long-term bevacizumab use given that one-third of patients will develop hypertension and proteinuria and that most patients will have hearing impairment. 

## Case presentation

A 38-year-old female previously diagnosed with NF-2 presented with chronic, progressive hearing loss and postural imbalance. She had bilateral vestibular schwannoma as well as supratentorial and spinal column meningioma. She had a translabyrinthine craniotomy and resection of vestibular schwannoma and instrumented cranial vault repair on the right side on July 10, 2017. This resulted in complete right-sided hearing loss. She was symptomatic in the left ear with significant tinnitus as well as subjective hearing loss. She was offered bevacizumab to preserve and maintain hearing in the left ear. Bevacizumab was given intravenously at a dose of 5 mg/kg of body weight every two weeks. Her blood pressure prior to starting treatment was 104/68 mmHg. At the time of writing, the patient had received 124 cycles of bevacizumab.

**Figure 1:**
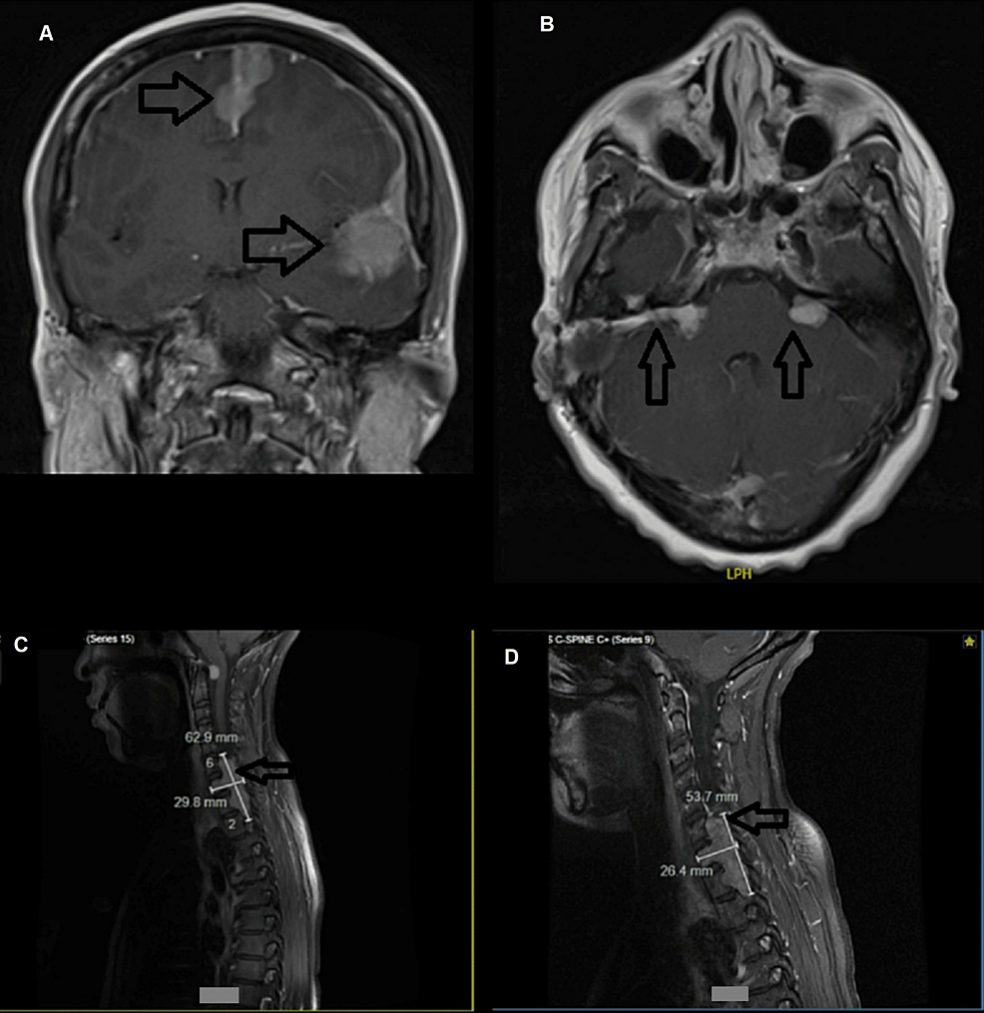
Gadolinium-enhanced T1 weighted MRI of the brain and spinal cord (A) Coronal sequence demonstrating parasagittal and left temporal homogenously enhancing lesions suggesting meningioma (arrows); (B) Axial sequence with characteristic bilateral vestibular schwannoma (arrows); (C) Sagittal sequence depicting a cervicothoracic extramedullary enhancing lesion prior to treatment with bevacizumab with dimensions of 62.9 x 29.8 mm (arrows); (D)Sagittal sequence depicting the same lesion seven months after treatment with bevacizumab with dimensions of 53.7 x 26.4 mm (arrows)

Within seven months of treatment, her tumor volumes decreased (Figure 1) and she reported significant hearing improvement in the left ear with and an associated reduction in tinnitus. However, she developed nephrotic range (3+ by urinalysis) proteinuria by her 93rd cycle of bevacizumab with normal creatinine. She developed hypertension while on bevacizumab in tandem with the onset of proteinuria with a blood pressure serially greater than 140/90 mmHg. Her bevacizumab treatment was held; however, she reported acute, severe hearing loss within one day of stopping bevacizumab and so it was restarted. She had an audiology assessment confirming hearing loss. Her hearing loss improved subjectively following the restart of treatment. An effort was made to get compassionate access to lapatinib to replace bevacizumab but this was declined by her insurer. She continued to have nephrotic range proteinuria intermittently while on bevacizumab so she was started an ARB, telmisartan by her 121st cycle. Previously she had been using a calcium channel blocker, amlodipine 5 mg daily, for hypertension. Two weeks following the start of telmisartan, she reported subjective improvement in hearing, resolution of proteinuria going from 3+ to 1+, and resolution of hypertension with her blood pressure normalizing to 135/85 mmHg.

## Discussion

NF-2 is a heritable disorder resulting in the eventual development of different tumor types affecting the nervous system including schwannoma, meningioma, and ependymoma.. Patients characteristically develop at least unilateral vestibular schwannoma, formerly known as acoustic neuroma, but commonly develop bilateral vestibular schwannoma. The development of bilateral vestibular schwannoma is one of the major diagnostic criteria for the disease and is present in 95% of cases. NF-2 has an incidence of one in 40,000 [1]. It is an autosomal dominant disorder resulting from a mutation in the NF2 tumor suppressor gene [2]. Typically, the tumors that arise from an NF-2 mutation are benign but they can impact quality of life and shorten lifespan to a degree. Hearing impairment and hearing loss represent the most common and significant source of impairment of quality of life in NF-2 patients [3]. NF-2 is diagnosed with neuroimaging and genetic testing [4]. Imaging features include homogeneously enhancing lesions representative of schwannoma in the bilateral internal acoustic canal extending from the vestibulocochlear nerve and dural-based, homogeneously enhancing lesions supratentorially and in the spinal column representative of meningioma as well as intraparenchymal lesions with heterogenous T2 and a gadolinium-enhancing signal representative of ependymoma [5].

Standard therapy for growing sporadic, unilateral vestibular schwannomas includes surgical removal or radiation therapy. Both treatments usually achieve tumor control, but at the frequent cost of hearing loss in the affected ear [6]. Only 41% of patients having stereotactic radiosurgery maintain their hearing at five years and attempts to completely resect these lesions typically result in leads to total hearing loss in the ear in almost all cases [7]. In recurrent cases, there is no standard of care. There is a paucity of randomized controlled trial (RCT) data for systemic therapy but many targeted therapies are currently in use including bevacizumab, lapatinib, and others [8]. Specifically, bevacizumab has been shown to reduce hearing loss and tumor volume in a prospective study by Plotkin et al. [9]. Patient-reported hearing questionnaires centered on hearing loss and hearing-related quality of life have been validated in adult and adolescent NF-2 populations lending support to patient reports of subjective hearing improvement throughout the disease course, absent formal audiology testing [10]. Long-term toxicity with the use of bevacizumab includes hypertension, bowel perforation, and poor wound healing [11]. 

A particular challenge is the development of nephrotic range proteinuria. This occurs through several pathomechanisms but a principal etiology is glomerular microangiopathy. It has not been shown conclusively to be a dose-dependent side effect. The incidence of proteinuria with bevacizumab in one study (n=42) was 35.7% with a median time to onset of 213 days. In the same study, the incidence of hypertension was 52.% with a median time to hypertension of 84 days [12]. ARB is used for renal protection in different renal disease states, including proteinuria and secondary hypertension [13,14]. A pre-clinical study has shown ARB to have a role in improving hearing loss in NF-2; specifically, losartan was shown to inhibit fibrogenic and inflammatory angiotensin signaling in a mouse model of NF-2 and showed that the treatment prevented hearing loss and normalized the tumor microenvironment by targeting IL6/STAT3 signaling [15]. Considering the possibility of a class effect with ARB, telmisartan is viewed as a potential agent for use concurrently with bevacizumab for the treatment of NF-2.

## Conclusions

This case report highlights the potential synergy with the concurrent use of ARB and bevacizumab in patients with NF-2. It suggests more research should be done to detail the mechanisms underlying tumor suppression and hearing improvement with this treatment strategy.
